# Lymphome primitif du sein: à propos d'un cas

**Published:** 2012-07-02

**Authors:** Noureddine Njoumi, Mohamed Najih, Laila Haqqi, Gilles Atolou, Abdessalm Bougtab, Hafid Hachi, Samir Benjelloun

**Affiliations:** 1 Service de chirurgie viscérale, HMIMV, Rabat, Maroc; 2 Service de chirurgie II, institut nationale d'oncologie, Rabat, Maroc

**Keywords:** Lymphome primitif, sein, diagnostic, traitement

## Abstract

Le lymphome primitif du sein est une entité histologique très rare du cancer du sein. Les aspects cliniques et radiologiques ne présentent pas de spécificités particulières. Le diagnostic est souvent retardé. Le traitement se base essentiellement sur la chimiothérapie. Le pronostic est globalement péjoratif. Nous rapportons un cas de lymphome malin non Hodgkinien primitif du sein chez une patiente de 38 ans. Parallèlement une revue de la littérature est entreprise évoquant les aspects épidémiologiques, cliniques, histologiques et thérapeutiques de ce néoplasme.

## Introduction

Le lymphome primitif mammaire (LPM) se définit par l'atteinte d'un ou des deux seins. Il s'agit du premier site atteint ou majoritairement atteint à l'exception d'une atteinte axillaire homolatérale.

La classification de Wiseman et Liao définit des critères diagnostiques de LPM: prélèvement histologique adéquat; étroite association entre le tissu mammaire et l'infiltration lymphomateuse; absence de diagnostic de lymphome extra mammaire et absence de métastases de la maladie excepté adénopathie axillaire homolatérale.

Cette néoplasie touche généralement la femme, cependant des cas chez l'homme ont été rapportés. C'est une affection rare, représentant 0,04–0,52% de tous les cancers du sein [1, 2]. Nous rapportons un nouveau cas de LPM colligé à l'institut national d'oncologie, la prise en charge de la patiente sera discutée à la lumière des données récentes de la littérature.

## Patient et cas Clinique

Il s'agit d'une patiente âgée de 38 ans, célibataire, sans antécédents personnels et familiaux notables. Le début de la maladie remonte à 03 mois par l'autopalpation d'un nodule du sein gauche qui a augmenté progressivement de volume, associé à une tension mammaire douloureuse, sans signes inflammatoires ni écoulement mamelonaire.

**Figure 1:**
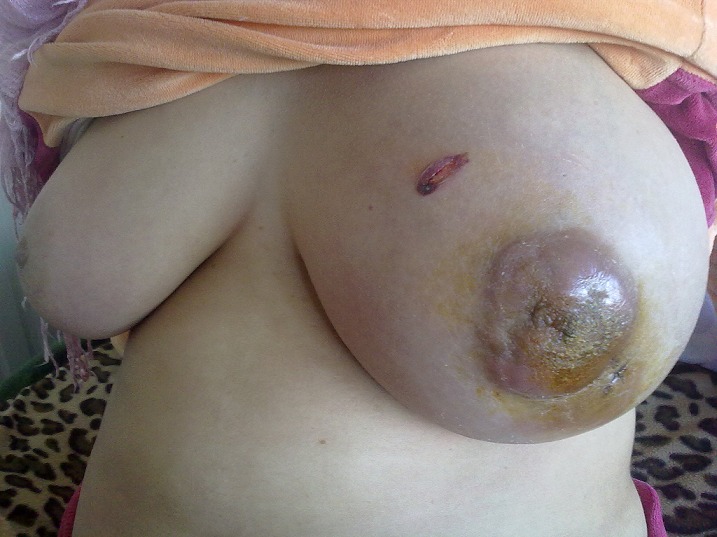
Gynécomastie de Lymphome primitif du sein gauche chez la patiente

L'examen du sein gauche trouve une gigantomastie avec rétraction mamelonaire (Figure 1). Une masse d'environ 10 cm de diamètre avec adénopathie axillaire homolatérale ont été retrouvées à la palpation. Le sein controlatéral et le reste de l'examen somatique sont normaux.

**Figure 2:**
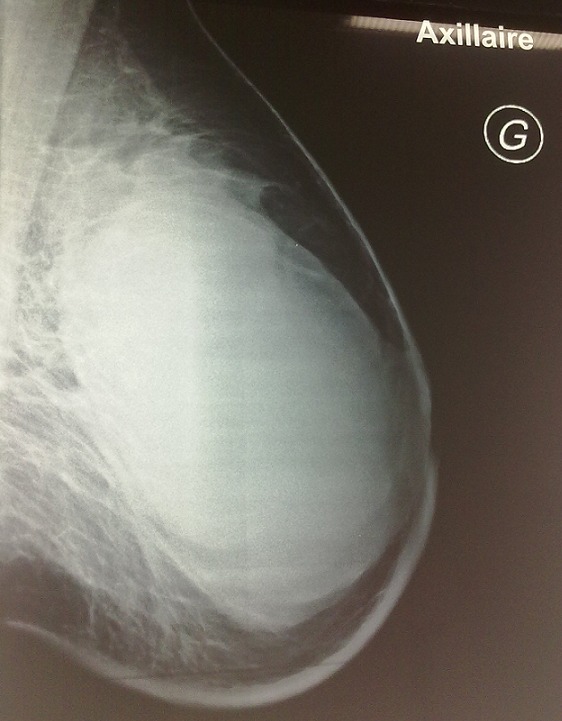
Mammographie du sein gauche montrant une importante opacité de tonalité hydrique

A la mammographie, on a mis en évidence des seins de type 3 en terme de densité mammaire avec volumineuse opacité de tonalité hydrique, relativement bien limitée, occupant la totalité de la masse mammaire gauche, mesurant 116 /80 mm.il y avait un épaississement cutanée et sous cutané mais sans foyer de microcalcification suspect (Figure 2).

Les deux seins sont classés ACR4 selon le système BIRADS. A l’échographie, l'opacité sus-décrite correspond à une formation tissulaire hypoéchogene, hétérogène hyper-vascularisée au doppler associée à de nombreuses adénopathies axillaires gauches avec épaississement de la peau et du tissu sous cutané.

**Figure 3:**
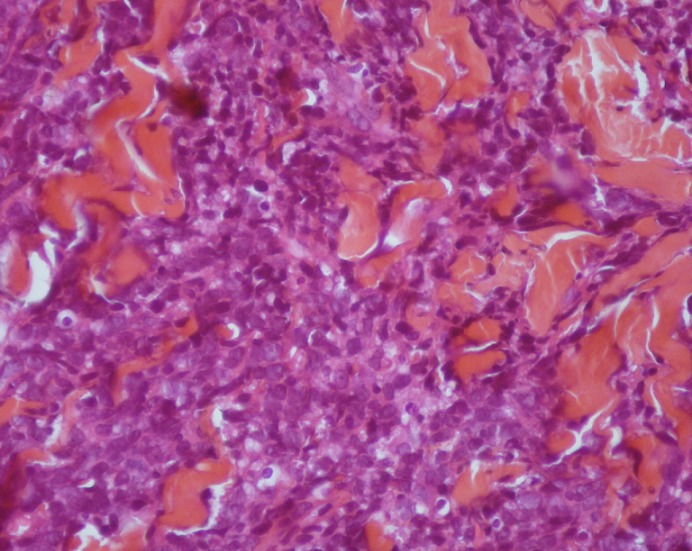
Coloration standard montrant à fort grossissement une prolifération cellulaire diffuse faite d’éléments de grande taille pourvus d'un cytoplasme peu abondant et mal délimité

**Figure 4:**
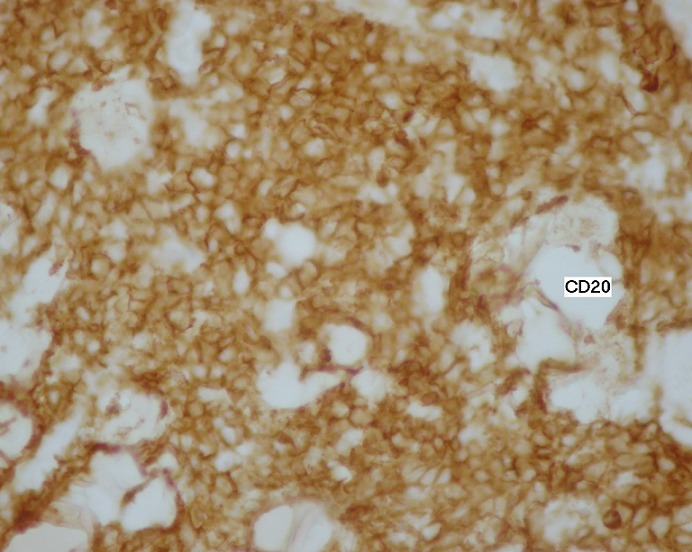
Etude immunohistochimique montrant à fort grossissement un marquage cellulaire diffus et intense par l'anti CD20, ces cellules sont négatives à l'anti CD3 et l'anti-cytokératine

L'examen histologique d'une biopsie chirurgicale du sein gauche montre un derme largement infiltré par un processus tumoral manifestement malin. Celui-ci se caractérise par une nappe cellulaire diffuse faite d’éléments de grande taille pourvus d'un cytoplasme peu abondant et mal délimité. Les noyaux sont arrondis ou ovalaires, hyperchromatiques et souvent nucléolés. Il existe quelques mitoses anormales (Figure 3). Ces cellules sont négatives pour l'anticorps anti-cytokératine (AE1-AE3). Elles sont négatives pour l'anticorps anti-CD 3 et franchement positives pour l'anticorps anti-CD20 (Figure 4). Le tout cadre avec un lymphome malin à grandes cellules B.

**Figure 5:**
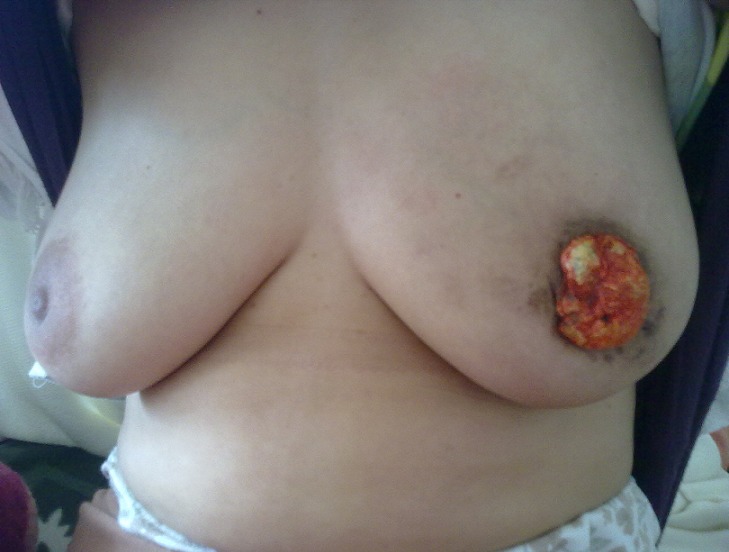
Aspect du sein à 5 mois d’évolution sous traitement

Un bilan d'extension comportant une TDM thoraco-abdominale et une biopsie ostéo-médullaire est revenu négatif. Une chimiothérapie à base de RCHOP a été décidée pour la patiente. Une amélioration clinique et radiologique a été obtenue après la 5^ème^ cure avec reduction de la taille tumorale de plus de 50% et disparaition des adénopathies axillaires (Figure 5). La patiente est en bon état général.

## Discussion

Les lymphomes primitifs du sein sont rares. Leur fréquence est estimée de 0,04 à 0,52% de tous les néoplasies du sein et 2,2% des lymphomes extra-nodaux [1, 2]. Cette pathologie touche généralement la femme, cependant des cas chez l'homme ont été rapportés. Concernant l’âge, deux pics de fréquence ont été notés, un premier pic chez la femme jeune en âge de procréation souvent au cours d'une grossesse, le second est plus important se situant entre 50 et 60 ans et de pronostic plus favorable [3].

L'atteinte est souvent unilatérale. Dans 18% des cas, elle est bilatérale, elle peut être simultanée (12%) ou successive (6%). Le mode de révélation est presque toujours le développement d'une tumeur mammaire [3, 4], très souvent aussi par une gigantomastie uni ou bilatérale avec un état de mastite inflammatoire [2] comme c’était le cas chez notre malade. Les adénopathies axillaires sont retrouvées dans 20 à 40% des cas [5].

L'aspect en imagerie est non spécifique. La mammographie montre souvent une masse bien limitée de densité homogène d'allure bénigne, évoquant un kyste, un fibro-adénome ou une tumeur phyllode. Moins fréquemment, il s'agit d'un aspect de mastite avec augmentation diffuse de la densité du sein, une masse de contours mal définis ou une masse à contours spiculés [6]. Rarement, on note un aspect suspect de malignité, mais il n'y a jamais d'opacité stellaire ni de microcalcifications [3]. En échographie la présentation n'est pas spécifique, le plus souvent sous forme d'une masse hypoéchogène homogène à contours nets et réguliers. Rarement un aspect de mastite est constaté en échographie. La discordance entre une clinique inquiétante et un aspect mammographique rassurant pourrait faire évoquer le diagnostic [3].

Le diagnostic est cytologique ou histologique après microbiopsie ou biopsie chirurgicale [7]. L’étude extemporanée comporte un risque d'erreur important, ainsi le diagnostic différentiel peut se poser avec les carcinomes anaplasiques ou les carcinomes médullaires, mais dans ces cas, le recours à l'immuno-histochimie permet de trancher devant l'absence d'expression des marqueurs épithéliaux (EMA, cytokératine) et l'immuno-expression des marqueurs lymphoïdes [8]. Le type histologique le plus fréquent est le lymphome diffus à grande cellules B. les lymphomes de bas grade de type MALT vient au deuxième rang par ordre d'incidence [2].

Le traitement du LMNH primitif du sein est superposable à celui des autres localisations lymphomateuses. De multiples protocoles ont été proposés dans la littérature [8]. Actuellement, la majorité des auteurs préconisent une chimiothérapie à base d'Endoxan^®^, Oncovin^®^ et Prednisone^®^ ou associée à une immunothérapie par anticorps anti-CD20.

Pour les LNH de haut grade de malignité ou à malignité intermédiaire, une polychimiothérapie seule est préconisée. Dans le cas o[ugrave] la tumeur est de taille inférieure à 5 cm, la chirurgie est d'abord réalisée. Dans le cas o[ugrave] la tumeur est volumineuse, la chirurgie pourra être précédée d'une chimiothérapie néo-adjuvante. Lorsque la chirurgie est impossible, une chimiothérapie sera associée à la radiothérapie. Le pronostic des LMNH du sein est particulièrement mauvais. Le type histologique et le stade clinique de la maladie sont les deux principaux facteurs pronostiques [3, 8].

## Conclusion

Le lymphome primitif du sein est une pathologie rare. Sa symptomatologie clinique est polymorphe. L'imagerie médicale est non spécifique. Le diagnostic de certitude est histologique. Le diagnostic d'un LPM impose un bilan d'extension soigneux. Le pronostic et le traitement rejoignent ceux des autres localisations lymphomateuses.
